# Injection of hybrid 3D spheroids composed of podocytes, mesenchymal stem cells, and vascular endothelial cells into the renal cortex improves kidney function and replenishes glomerular podocytes

**DOI:** 10.1002/btm2.10212

**Published:** 2021-01-21

**Authors:** Wen‐Yu Yang, Li‐Chi Chen, Ya‐Ting Jhuang, Yu‐Jie Lin, Pei‐Yu Hung, Yi‐Ching Ko, Meng‐Yu Tsai, Yun‐Wei Lee, Li‐Wen Hsu, Chih‐Kuang Yeh, Hsiang‐Hao Hsu, Chieh‐Cheng Huang

**Affiliations:** ^1^ Institute of Biomedical Engineering National Tsing Hua University Hsinchu Taiwan; ^2^ Department of Biomedical Engineering and Environmental Science National Tsing Hua University Hsinchu Taiwan; ^3^ Kidney Research Center, Department of Nephrology Linkou Chang Gung Memorial Hospital Taoyuan Taiwan; ^4^ Bioresource Collection and Research Center Food Industry Research and Development Institute Hsinchu Taiwan; ^5^ College of Medicine, School of Medicine Chang Gung University Taoyuan Taiwan

**Keywords:** cell spheroids, cell therapy, glomerulus, kidney injury, podocyte

## Abstract

Podocytes are highly differentiated epithelial cells that are crucial for maintaining the glomerular filtration barrier in the kidney. Podocyte injury followed by depletion is the major cause of pathological progression of kidney diseases. Although cell therapy has been considered a promising alternative approach to kidney transplantation for the treatment of kidney injury, the resultant therapeutic efficacy in terms of improved renal function is limited, possibly owing to significant loss of engrafted cells. Herein, hybrid three‐dimensional (3D) cell spheroids composed of podocytes, mesenchymal stem cells, and vascular endothelial cells were designed to mimic the glomerular microenvironment and as a cell delivery vehicle to replenish the podocyte population by cell transplantation. After creating a native glomerulus‐like condition, the expression of multiple genes encoding growth factors and basement membrane factors that are strongly associated with podocyte maturation and functionality was significantly enhanced. Our in vivo results demonstrated that intrarenal transplantation of podocytes in the form of hybrid 3D cell spheroids improved engraftment efficiency and replenished glomerular podocytes. Moreover, the proteinuria of the experimental mice with hypertensive nephropathy was effectively reduced. These data clearly demonstrated the potential of hybrid 3D cell spheroids for repairing injured kidneys.

## 1 INTRODUCTION

The kidney glomerulus, which is composed of podocytes, capillary endothelial cells, mesangial cells, and the glomerular basement membrane (GBM), is the apparatus for blood filtration and urine production.^1^, ^2^ As highly differentiated epithelial cells, podocytes form interdigitating foot processes and wrap the GBM together with glomerular capillaries to serve as a filtration barrier that prevents protein loss,^3^, ^4^ while mesangial cells are specialized pericytes that are localized adjacent to the vascular endothelium and support the normal function of the glomerulus by secreting multiple factors.^5^ Podocytes are active in secreting growth factors and hormones, such as vascular endothelial growth factor A (VEGFA) that is essential for maintaining the endothelial integrity of the glomerular filtration barrier.^6^ It is now well recognized that podocyte injury followed by depletion and the resultant proteinuria is the major cause of the initiation and pathological progression of multiple types of nephropathy.^7^, ^8^ Once podocyte loss surpasses a certain threshold, the development of glomerulosclerosis and subsequent end‐stage kidney failure is almost inevitable.^3^, ^7^

Despite the global pandemic of kidney disease, current therapeutic options are extremely limited. The mainstream pharmaceutical‐based approaches often fail to effectively arrest or reverse the progression of nephropathy.^3^ Kidney transplantation and dialysis are the ultimate life‐saving options for patients who develop renal failure.^9^ However, kidney transplantation is severely hindered by the shortage of donors, while dialysis significantly diminishes the patients' quality of life,^3^, ^9^, ^10^ and the multiple accompanying life‐threatening complications are also significant issues. Therefore, patients with kidney disease desperately need a new therapeutic solution that can arrest disease progression or even restore impaired kidney function.

Accumulating evidence suggests that the magnitude of podocyte depletion may predict future progression and the extent of glomerulosclerosis.^7^, ^8^, ^11^ Therefore, strategies that can inhibit podocyte loss and restore their number may potentially become novel therapies for curing kidney diseases. Nevertheless, adult podocytes are postmitotic cells that are unable to proliferate under physiologic conditions.^1^ Although parietal epithelial cells may serve as progenitors and differentiate into podocytes,^12^, ^13^ the number of newly generated podocytes fails to compensate for the depleted number of cells and achieve renal functional improvement.

Current advances in stem cell technology and engineering may provide a new means of cell replacement therapy to replenish podocyte loss. Ahmadi et al reported that transplantation of induced pluripotent stem cell‐derived podocytes into the renal cortex parenchyma ameliorated glomerular fibrosis and restored kidney function.^14^ However, the podocytes were maintained under two‐dimensional (2D) conditions and thus lacked the three‐dimensional (3D) context that mimics the microenvironment of the native glomeruli. Additionally, conventional cell transplantation approaches employ trypsin and saline as cell dissociation agents and cell delivery vehicles, respectively. In such scenarios, podocytes may lack sufficient cell–cell, cell–extracellular matrix (ECM), or cell–GBM interactions, thus facilitating detachment‐induced anoikis.^15^, ^16^, ^17^ Moreover, the administered cell suspensions tend to leak out or reflux from the duct of injection, thus causing the loss of engrafted cells in the target area.^17^, ^18^

To address the abovementioned issues, this study aims to transplant podocytes in the configuration of multicellular 3D spheroids, which are known to recapitulate the in vivo physiological conditions^19^, ^20^ and improve the cell retention and survival after injection.^21^, ^22^ As reported by our group^23^, ^24^, ^25^ and other groups,^26^, ^27^ 3D cell spheroids exhibit enhanced intercellular interactions, a well‐preserved ECM, and an increased physical size compared to those of single cells, and can be harvested without the use of proteolytic enzymes, thus benefiting cellular function and the ultimate therapeutic outcome of cell therapy.

As neighboring cells and microenvironments are crucial for modulating cellular behaviors, we further developed hybrid 3D cell spheroids composed of three cell types: podocytes, human umbilical vein endothelial cells (HUVECs), and mesenchymal stem cells (MSCs). HUVECs were used to simulate glomerular endothelial cells, while MSCs have been demonstrated to be able to differentiate into mesangial‐like cells.^28^ As a result, we anticipated that the presence of HUVECs and MSCs in hybrid 3D cell spheroids may help establish a native glomerulus‐mimicking niche for podocytes, thereby enhancing their cellular function and postengraftment integration with host podocytes in the glomeruli.

Herein, hybrid 3D cell spheroids composed of podocytes (P), MSCs (M), and HUVECs (E) were prepared using a methylcellulose (MC) hydrogel system reported in our previous publications.^21^, ^23^, ^29^ Spheroids with two (PM and PE) or one (P only) cell type served as controls. By exploring the mutual relationships among these cell types, we found that the maturation and engraftment of the administered podocytes were further strengthened by their interaction with the surrounding MSCs and HUVECs. Moreover, transplantation of hybrid 3D cell spheroids via intrarenal administration in a mouse model of hypertensive nephropathy preserved the glomerular podocyte number and improved renal function. Therefore, we anticipated that podocyte transplantation using the developed hybrid 3D cell spheroids may hold great potential for enhancing the efficiency of podocyte replenishment and subsequent therapeutic efficacy.

## 2 RESULTS

### 2.1 Podocytes, MSCs, and HUVECs assemble into hybrid 3D cell spheroids

**FIGURE 1:**
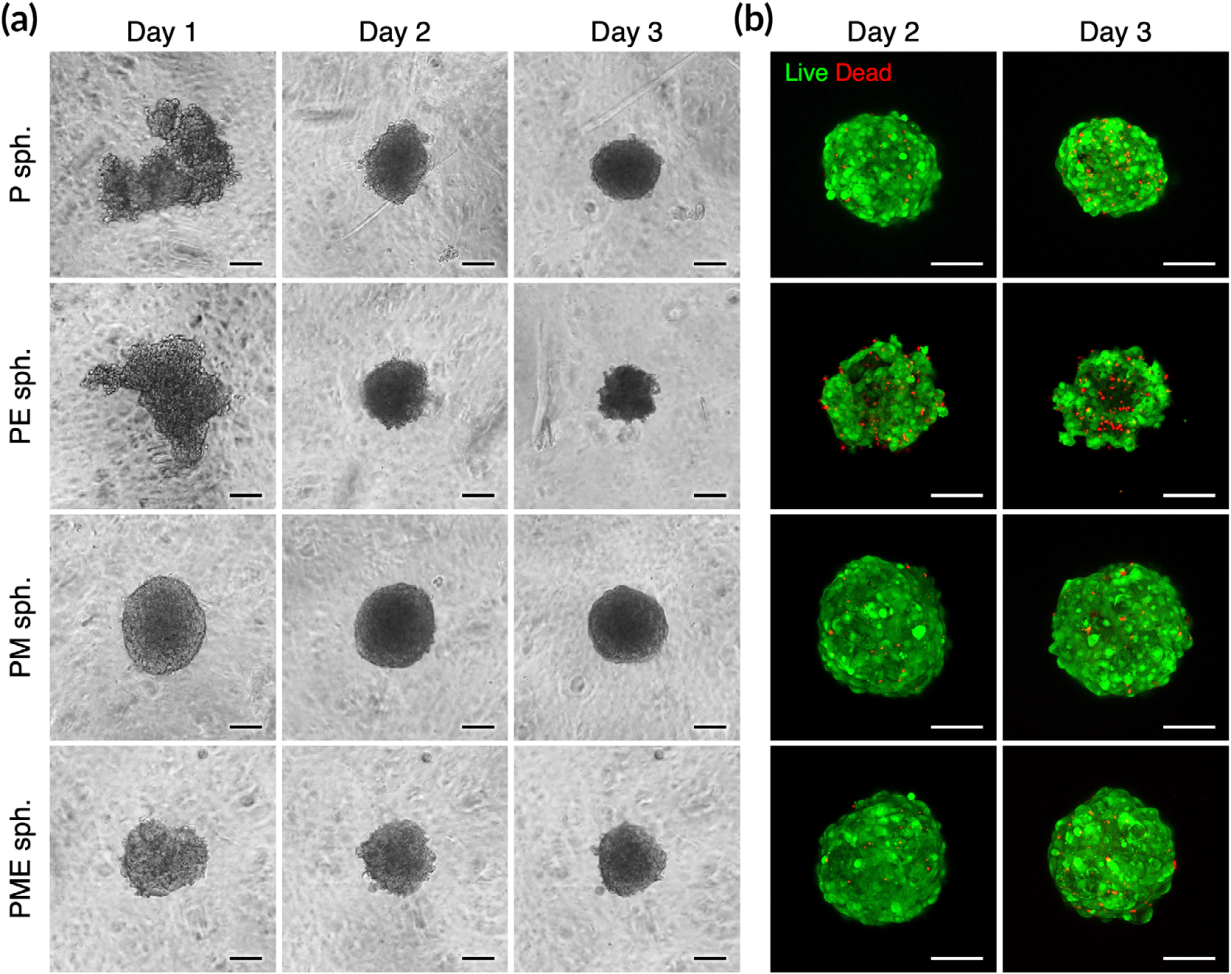
Podocytes (P), mesenchymal stem cells (M), and vascular endothelial cells (E) assemble into hybrid three‐dimensional (3D) cell spheroids (sph.). Representative (a) phase‐contrast photomicrographs and (b) live/dead fluorescence images of the fabricated 3D cell spheroids. Scale bars, 100 μm

To assemble cells into the 3D spheroid configuration, cell suspensions with the desired cell types and cell numbers were added into a 96‐well plate precoated with MC hydrogel. After a 24 h culture, the cells of the PM and PME groups were condensed into spherical aggregates in each well (Figure 1(a)). For the P and PE groups, however, the cells in each well clumped loosely with an irregular morphology, which was not suitable for further manipulation (Figure 1(a)). By prolonging the incubation period to 48 or 72 h, one 3D cell spheroid was formed in each well for all groups (Figure 1(a)). We next assessed the optimal culture period for cell spheroid fabrication by investigating cell viability using live/dead staining. As revealed by the fluorescence images in Figure 1(b) and Figure S1, except for the PE spheroids, no obvious cell death was observed at Day 2. As time progressed to Day 3, however, more dead cells were detected in all groups. Therefore, cell spheroids that were fabricated with a 2‐day incubation were chosen for the following studies.

**FIGURE 2:**
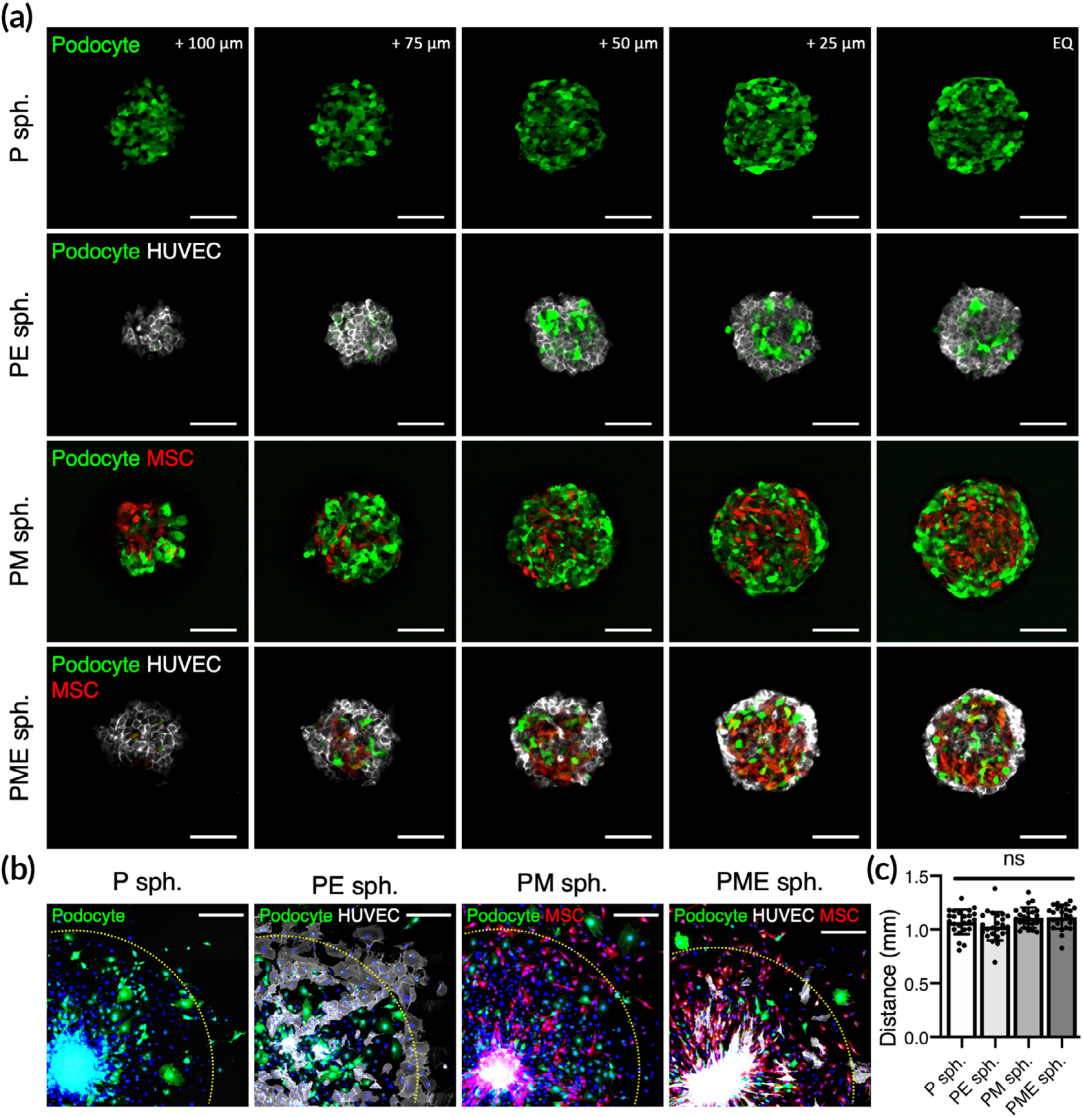
(a) Confocal Z‐stack images showing the composition of multiple cell types in hybrid 3D cell spheroids. Scale bars, 100 μm. (b) Fluorescence images of podocytes that migrated from the attached spheroids and (c) the distance that the podocytes traveled (*n* = 24 pooled from three different cell spheroids). Scale bars, 300 μm. Yellow dashed lines indicate 1 mm from the center of the attached cell spheroids. ns, not significant

The average diameters of the harvested P, PE, PM, and PME spheroids were 245.4 ± 10.1, 230.8 ± 17.2, 261.5 ± 9.8, and 241.4 ± 10.3 μm, respectively (*n* = 3 batches). We next sought to assess the distribution of each cell type within the 3D spheroids using confocal microscopy. Herein, podocytes that expressed green fluorescence protein (GFP) and MSCs that expressed red fluorescence protein (RFP) were used in this study, while HUVECs were identified by immunofluorescence staining against CD31. Figure 2(a) shows the confocal image Z‐stacks of the fabricated hybrid 3D cell spheroids at various distances from the equatorial section, demonstrating the incorporation of multiple cell types into the multicellular architecture. To investigate whether the migration of podocytes from spheroids is affected by the presence of MSCs or HUVECs, 3D cell spheroids were harvested, plated in culture plates, and incubated for 3 days. As shown in Figure 2(b) and (c), the longest distances that podocytes traveled were comparable among all of the experimental groups, suggesting that the presence of MSCs and HUVECs neither promotes nor impedes the migration of podocytes.

### 2.2 Mutual interactions among cells within 3D spheroids enhance cellular functionality

We next investigated the differences in podocyte behaviors in 2D or 3D configurations and in the presence of MSCs or HUVECs. As the conditionally immortalized mouse podocytes were maintained at 33°C in the presence of interferon (IFN)‐γ (permissive condition) and induced to differentiate at 38°C without IFN‐γ (nonpermissive condition), we first evaluated the expression profile of the differentiation‐related gene *Synpo* in podocytes exposed to various configurations and neighboring cells using real‐time quantitative polymerase chain reaction (PCR).

**FIGURE 3:**
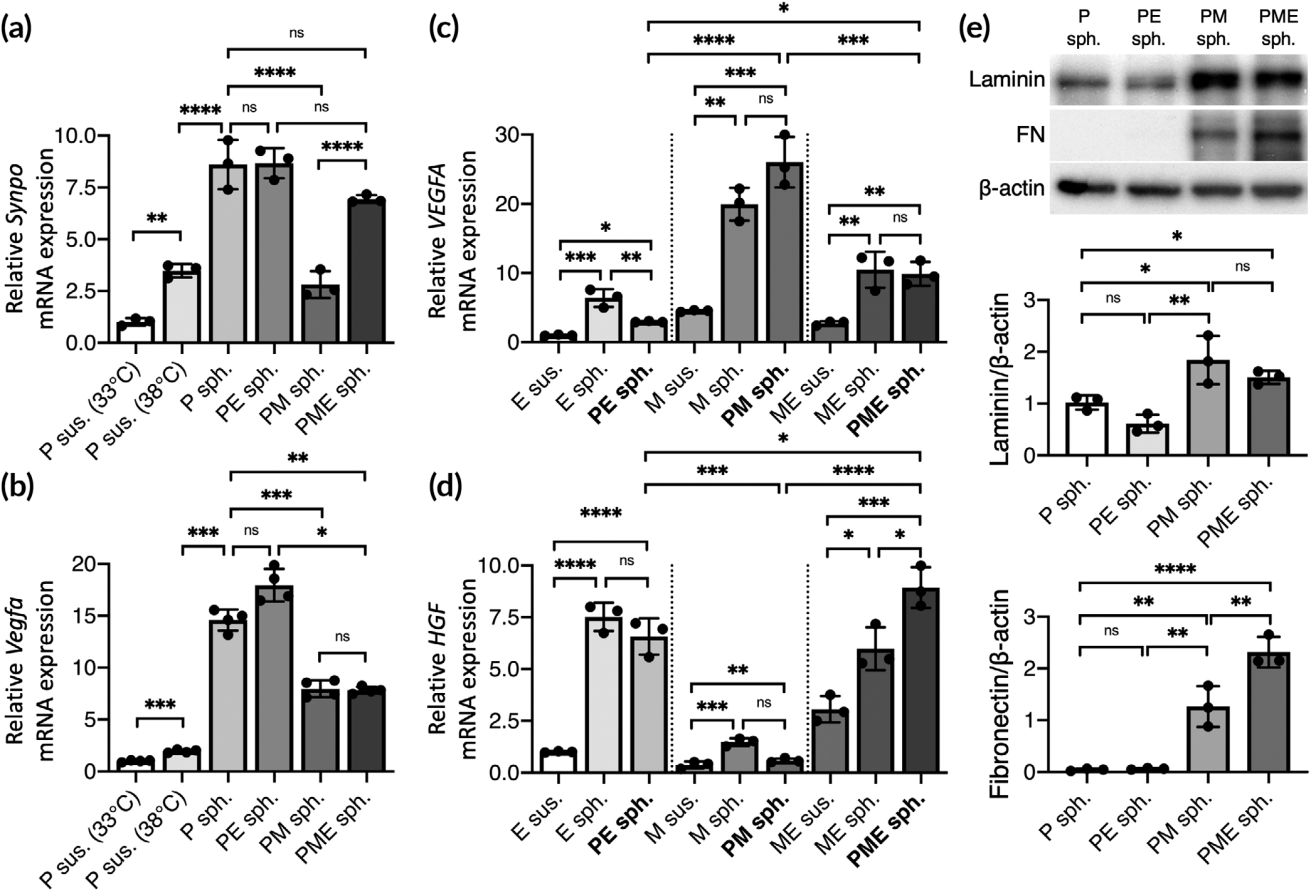
Mutual interactions among cells within 3D spheroids enhance cellular functionality. Expression levels of mouse (a) *Synpo* and (b) *Vegfa* and human (c) *VEGFA* and (d) *HGF* mRNA in hybrid 3D cell spheroids determined by real‐time quantitative PCR. The results are expressed as the fold change relative to the control podocytes (*n* = 3). (e) Western blot for laminin, fibronectin (FN) and β‐actin in hybrid 3D cell spheroids and the corresponding quantitative results normalized to β‐actin (*n* = 3). Data are presented as mean ± SD. All *p* values were calculated using one‐way ANOVA with the Tukey correction. *p < 0.05; **p < 0.01; ***p < 0.005; ****p < 0.001; ns, not significant

Elevation of the culture temperature induced *Synpo* expression (3.5‐fold increase compared with the control; *p* < 0.01; Figure 3(a)), which could be further enhanced significantly by assembly into 3D P spheroids (8.6‐fold increase compared with control; *p* < 0.0001). Additionally, compared with the P spheroid group, the incorporation of HUVECs into the spheroids (PE spheroids) did not alter the expression profile of *Synpo* (*p* > 0.05 compared with P spheroids), while MSCs in PM spheroids tended to delay the progression of podocyte differentiation (*p* < 0.001 compared with P spheroids). In the PME spheroid group, however, the level of *Synpo* expression was maintained as seen in the P spheroid group (*p* > 0.05 compared with P spheroids).

We next sought to analyze the mRNA expression profiles of *Vegfa* and *VEGFA*. After cultivation under nonpermissive conditions in a 2D configuration for 2 days, the mouse podocytes exhibited slightly increased *Vegfa* expression compared with that of the control (1.9‐fold increase; *p* < 0.005; Figure 3(b)). When assembled into the 3D conformation, however, a significant elevation in the level of *Vegfa* expression was observed (14.6‐fold increase compared with the control; *p* < 0.001). Although the enhancement of mouse *Vegfa* mRNA expression observed in P spheroids was weakened in the PME spheroid group (7.8‐fold increase compared with the control; *p* < 0.005; Figure 3(b)), it might be compensated by the increased expression of the human *VEGFA* gene by HUVECs and MSCs in PME spheroids (3.6‐fold increase compared with cell suspensions of MSCs and HUVECs; *p* < 0.01; Figure 3(c)). Therefore, the assembly of podocytes, MSCs and HUVECs into the 3D spheroid configuration benefits their expression of mouse and human VEGFA proteins.

We also investigated the response of MSCs and HUVECs to podocytes in terms of the expression of hepatocyte growth factor (HGF). Although the presence of podocytes in the PE or PM spheroids did not alter the expression of *HGF* mRNA by MSCs or HUVECs (*p* > 0.05; Figure 3(d)), the podocytes incorporated in the PME spheroids exhibited significantly increased overall *HGF* expression (1.5‐fold increase compared with 3D spheroids of MSCs and HUVECs; *p* < 0.05; Figure 3(d)).

We next analyzed the deposition of laminin and fibronectin, two of the major components of the GBM, in the fabricated 3D cell spheroids. In the PE spheroid group, the levels of laminin and fibronectin were similar to those in the P spheroid group (*p* > 0.05; Figure 3(e)). Conversely, the presence of MSCs in the PM spheroid group resulted in a significant increase in the laminin and fibronectin content in 3D cell spheroids (1.8‐ and 25.2‐fold increases in laminin and fibronectin content, respectively, compared with P spheroids; *p* < 0.05; Figure 3(e)). Moreover, the level of fibronectin in 3D cell spheroids could be further elevated by simultaneous incorporation of MSCs and HUVECs into the PME spheroids (1.9‐fold increase compared with the PM spheroids; *p* < 0.01; Figure 3(e)). In summary, in terms of podocyte differentiation, growth factor secretion, and matrix deposition, the PME spheroids exhibited potential superior to that of the other studied groups and were thus chosen for subsequent therapeutic application.

### 2.3 3D cell spheroids contain abundant ECM proteins and growth factors

**FIGURE 4:**
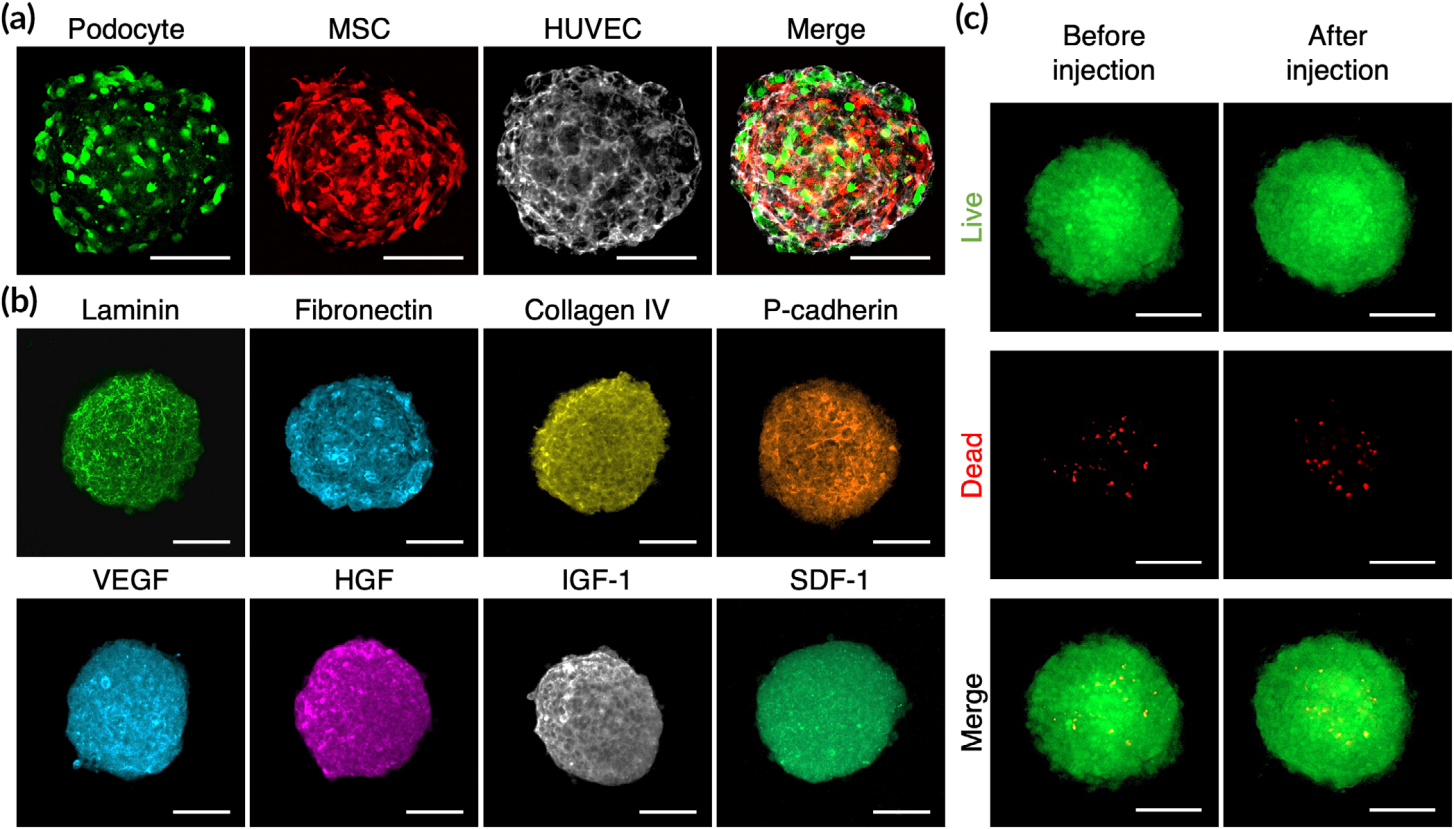
3D cell spheroids contain abundant extracellular matrix (ECM) proteins and growth factors. Fluorescence images showing (a) the presence of three cell types and (b) the inherent extracellular matrix and growth factors within hybrid 3D cell spheroids. (c) Representative live/dead images of 3D cell spheroids before and after injection through a needle. Scale bars, 100 μm

Confocal images acquired from the equatorial plane of the PME spheroids revealed the cellular compositions within the 3D structures (Figure 4(a)). As these 3D cell spheroids were collected without trypsinization, ECM proteins including laminin, fibronectin and collagen IV that were deposited by the cells were well retained (Figure 4(b)). Additionally, cell adhesion molecules such as P‐cadherin were detected, demonstrating extensive cell–cell contacts and interactions in the harvested 3D spheroids (Figure 4(b)). Moreover, the fabricated spheroids contained numerous growth factors that were produced by cells (Figure 4(b)) and thus hold great potential for the engineering of a pro‐proliferative microenvironment for the engrafted cells.

We next further investigated whether the transplantation of 3D cell spheroids via injection resulted in harmful effects on the delivered cells. The cell spheroids were loaded into a syringe and injected through a 24‐gauge needle. The viability of the cells within the spheroids was assessed using live/dead staining. As indicated by the fluorescence images, the difference in cell viability between the control and injected spheroids was not significant (Figure 4(c)), demonstrating that the majority of the cells survived the administration procedures.

### 2.4 Cell transplantation into the renal cortex using a 3D spheroid configuration enhances cell retention

**FIGURE 5:**
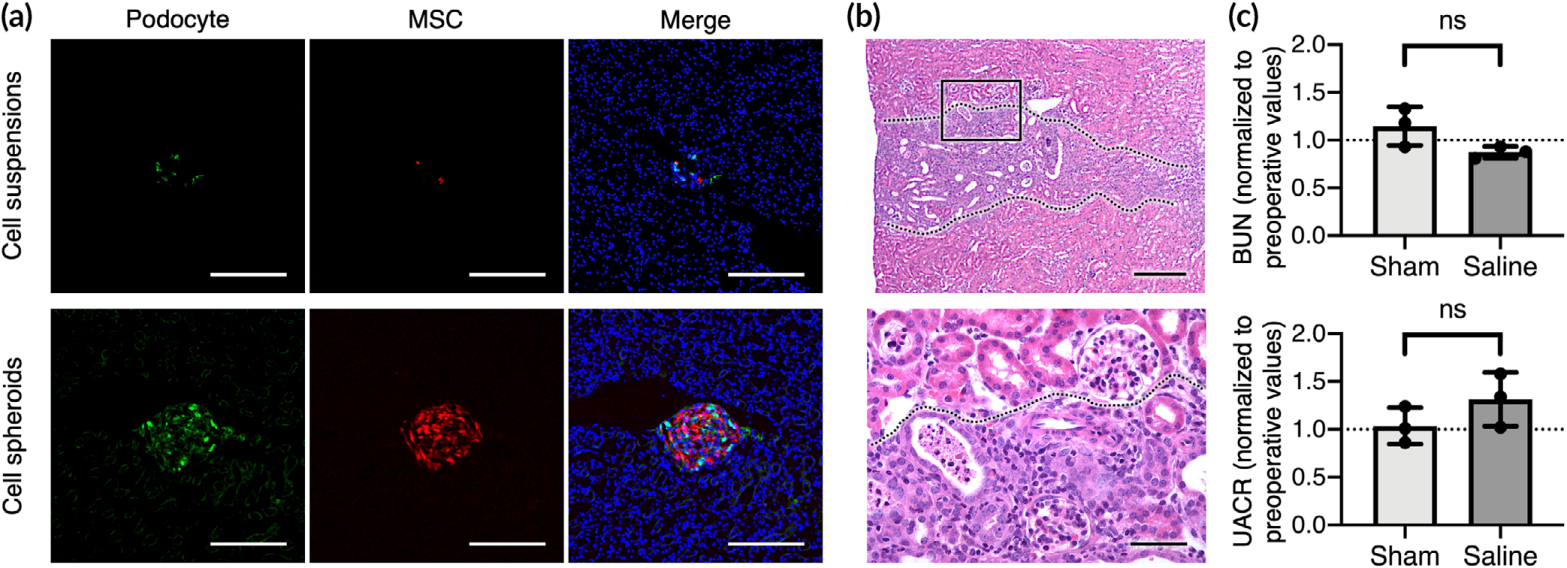
Direct injection into the renal cortex does not result in significant kidney injury. (a) Representative fluorescent images showing the transplanted cells in kidney. Scale bars, 200 μm. (b) Results of hematoxylin and eosin (H&E) staining showing the injection path (indicated by dotted lines). Scale bars, 200 μm (upper panel) or 50 μm (bottom panel). (c) The blood urea nitrogen (BUN) and urine albumin‐creatinine ratio (UACR) of healthy experimental animals at Day 15 after treatment are normalized to preoperative values (*n* = 3). Data are presented as mean ± SD. The *p* values were calculated using two‐tailed Student's *t*‐test. ns, not significant

The developed PME spheroids were first injected intrarenally into healthy mice. At 1 h after administration, the kidneys were retrieved, cryosectioned, and processed for the detection of the engrafted cells. As revealed in the fluorescence images, the GFP‐positive podocytes and RFP‐positive MSCs delivered in the 3D spheroid conformation were easily observed at the injection site, whereas the cells administered in cell suspensions were occasionally detected (Figure 5(a)).

To evaluate whether the hydrodynamic force generated during injection cause detrimental effects on the kidney, the renal structure and function of the experimental animals were investigated at 15 days postoperation. As indicated by the results of hematoxylin and eosin staining, small areas of inflammatory cell infiltration were observed in the renal cortex of the injection path in the animals that were injected with 3D cell spheroids (Figure 5(b)). However, the levels of blood urea nitrogen (BUN) and the urine albumin‐creatinine ratio (UACR) of the mice that received 3D cell spheroids injection at Day 15 remained unchanged compared to that before operation for the same group (*p* > 0.05; Figure 5(c)), suggesting that direct renal cortex injection might result in only limited kidney injury.

### 2.5 Transplantation of hybrid 3D cell spheroids improves renal function in mice with hypertensive nephropathy

**FIGURE 6:**
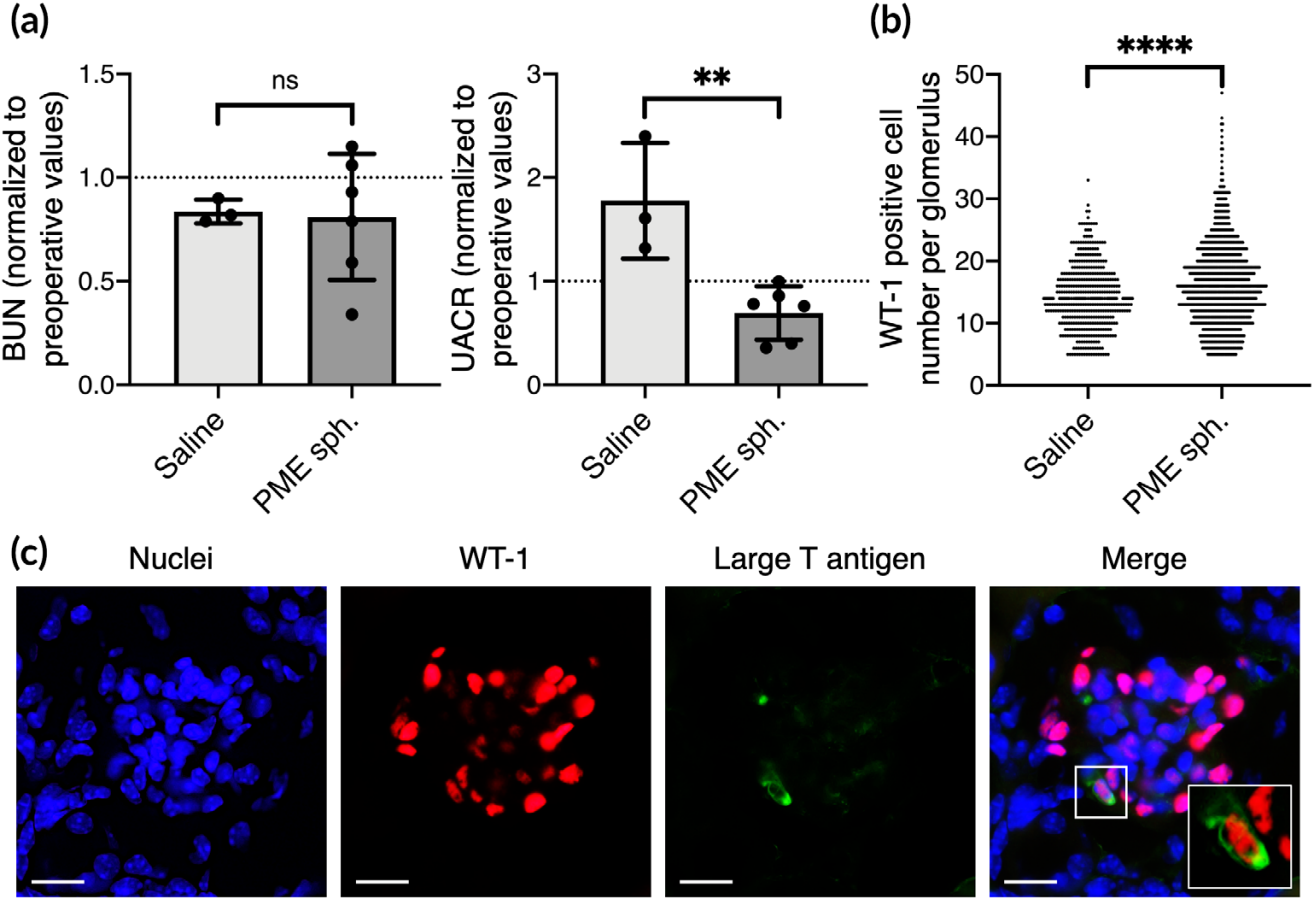
Transplantation of hybrid 3D cell spheroids improves renal function in mice with hypertensive nephropathy. (a) The blood urea nitrogen (BUN) and urine albumin‐creatinine ratio (UACR) of experimental animals that received saline (*n* = 3) or PME spheroids (n = 6) are normalized to preoperative values. (b) Number of podocytes per glomerulus of the kidney retrieved from the experimental animals. (c) Fluorescence images showing the engraftment of transplanted large T antigen‐positive podocytes. Inset shows a magnified view. Scale bars, 10 μm. All *p* values were calculated using two‐tailed Student's *t*‐test. **p < 0.01; ****p < 0.001; ns, not significant

Mice with genetically induced hypertension and associated hypertensive nephropathy were administered saline or PME spheroids. Although the changes of BUN levels were not obvious, the level of UACR in animals that received PME spheroids decreased significantly compared to that of animals that received saline at Day 15, suggesting reduced proteinuria and improved glomerular permselective function (*p* < 0.01; Figure 6(a)). The observed beneficial effects were associated with a significant increase in the number of glomerular podocytes, identified by Wilms tumor 1 (WT1) immunohistochemistry (Figure S2), in the PME group (16.3 ± 6.9 WT1‐positive cells per glomerulus) compared to the saline‐treated group (14.2 ± 5.3 WT1‐positive cells per glomerulus; *p* < 0.001; Figure 6(b)). Finally, the results of immunofluorescence staining confirmed the presence of large T antigen‐positive podocytes in the glomeruli, suggesting the incorporation and engraftment of transplanted podocytes (Figure 6(c)).

## 3 DISCUSSION

Cell therapy has been exploited for treating kidney injury by replenishment or replacement of desired cell types, such as podocytes,^14^, ^30^ or by engineering a pro‐regenerative microenvironment.^31^, ^32^ Herein, we successfully demonstrated that the assembly of podocytes into a 3D spheroid configuration could efficiently enhance their maturation and cellular function. Moreover, after the incorporation of MSCs and HUVECs, the podocytes within the engineered hybrid 3D cell spheroids exhibited further strengthened functionality, which can be attributable to the enhanced cell–cell interactions among the heterogeneous cell populations within the spheroids.

To fabricate 3D spheroids, cells were plated into MC hydrogel‐coated 96‐well plates and incubated for predetermined periods. As reported in our previous publications,^21^, ^23^ the MC hydrogel provides a nonattachable surface for the seeded cells, thus prompting the aggregation of anchorage‐dependent cells. It seems that 3D spheroids without MSCs (P and PE spheroids) adopted a relatively irregular architecture on the first day, followed by concentration into the spheroid configuration, whereas spheroids containing MSCs (PM and PME spheroids) formed a spherical morphology at 1 day after plating, suggesting that the presence of MSCs was able to promote the aggregation of cells. MSCs have been reported to secrete a substantial amount of ECM to promote the cell–cell and cell–ECM interactions during the formation spheroid architecture,^33^ thus increasing the compactness of spheroids that are composed of multiple cell types.^33^, ^34^, ^35^ As 3D spheroids inherently generate gradients of oxygen, nutrients, and metabolic waste,^36^ it is not surprising that extended incubation led to increased cell death within the spheroids.

Podocytes and vascular endothelial cells are known to establish the filtration barrier in the glomeruli.^1^ Additionally, it has been reported that an MSC‐like population exists in glomerular and tubular compartments and may contribute to injury repair.^37^ As the maintenance of normal podocyte structure and function is highly modulated by complicated cell–cell interactions through direct juxtaposition or indirect cytokine stimulation,^38^ the involvement of MSCs and HUVECs within the 3D spheroids could more accurately mimic the microenvironment found in the glomerulus than the spheroids that were composed of only podocytes, as evidenced by the enhanced efficiency of podocyte maturation. It has been reported that different cell types may self‐organize to form heterogeneous populations with 3D spheroids.^39^ Moreover, organoids formed by stem cells may develop into a native glomerulus‐like architecture, which is characterized by an involuted podocyte layer with a capillary loop.^4^ In the present study, however, we did not observe specific patterns within the PME spheroids, probably because the spherical configuration was only maintained for 2 days.

Podocytes reportedly produce a large amount of VEGFA protein that is crucial for maintaining the survival of podocytes and the integrity of the filtration barrier.^2^ In addition, the potential of HUVECs and MSCs to secrete VEGFA has been well documented. By establishing hybrid 3D cell spheroids, the expression of both human *VEGFA* and mouse *Vegfa* genes was significantly upregulated, thus demonstrating the advantage of hybrid 3D cell spheroids in promoting the functionality of podocytes. Moreover, the significantly enhanced expression of *HGF* mRNA by HUVECs or MSCs in PME spheroids might have a beneficial effect for podocyte transplantation, as HGF has been demonstrated to protect podocytes from apoptosis and prevent kidney fibrosis.^40^

In addition to paracrine secretion, the introduction of HUVECs or MSCs also increased the amount of laminin and fibronectin, two of the ECM components that constitute the GBM, deposited by the cells in 3D spheroids, thus creating a native glomerulus‐like microenvironment. Although the capacity of MSCs in the 3D spheroid configuration to secrete fibronectin and laminin has been reported in the literature,^41^, ^42^ the reason for the observed enhancement of ECM component secretion upon exposure to both podocytes and HUVECs remains unclear in the present study. Therefore, further investigations regarding the mutual interactions among the three employed cells types are warranted to elucidate the underlying mechanisms. The results of immunostaining further confirmed the presence of abundant ECM molecules within the collected hybrid 3D cell spheroids, which thus hold great potential for preventing anoikis‐induced cell death during cell harvest or transplantation. Moreover, as the ECM can function as an effective reservoir to store and protect growth factors,^43^ the matrix proteins inherently present in 3D cell spheroids may benefit the retention and activity of the deposited growth factors. When injected into the renal cortex, the transplanted cells that were preassembled into the 3D spheroid configuration were actually codelivered with the associated ECM and soluble factors, which might contribute substantially to the survival and function of engrafted podocytes.

Direct injection into the renal cortex has been reported to induce transient kidney injury that can be resolved within 2 weeks.^43^ In line with the observed phenomenon in the literature, our results demonstrated that a single intrarenal injection of saline had no effect on the overall kidney function, although slight tissue fibrosis was found along the track of the injecting needle. Notably, cell transplantation in this study was performed without immunosuppression, thus allowing host immune cells to reject xenograft human MSCs and HUVECs.^44^ Nevertheless, their initial presence efficiently enhanced podocyte viability and functionality, thus eventually contributing to an increase in the overall therapeutic efficacy. Moreover, MSCs have been recognized for their immunomodulatory potential.^45^, ^46^, ^47^ Therefore, the anti‐inflammatory action exerted by MSCs on local renal tissues might also contribute to the improved kidney function.^48^, ^49^ Further work is necessary to elucidate the detailed mechanisms among the engrafted podocytes and MSCs as well as the host glomeruli and renal inflammatory microenvironment. Another limitation in the present study is that although the transplanted podocytes were observed to be located in mouse glomeruli, these cells might not be necessary to form functional filtration slits together with host podocytes. As indicated by our in vitro results, the hybrid 3D cell spheroids exhibited significantly enhanced paracrine secretion and matrix deposition, both of which might potentially modulate the local microenvironment in the target renal tissue. Therefore, further studies are warranted to verify the functionality of the implanted cells and the mechanisms of the observed therapeutic benefits.

## 4 CONCLUSION

Taken together, this proof‐of‐principle study showed that by engineering hybrid 3D cell spheroids containing podocytes, MSCs and HUVECs, a glomerulus‐mimicking microenvironment could be established to enhance the maturation and functionality of podocytes. The hybrid 3D cell spheroids contained abundant ECM and growth factors, thus benefiting the viability and therapeutic potential of the postengrafted podocytes. Transplantation of the hybrid 3D cell spheroids into the renal cortex improved the kidney function of mice with hypertensive nephropathy, indicating that podocyte transplantation with the developed hybrid 3D cell spheroids may hold great potential to further enhancing the efficiency of podocyte replenishment and subsequent therapeutic function.

## 5 MATERIALS AND METHODS

### 5.1 Cell culture

Conditionally immortalized podocytes that carry the temperature‐sensitive SV40 large T antigen, derived from Endlich's group,^50^ were cultivated in RPMI 1640 medium (Thermo Fisher Scientific, Waltham, MA) supplemented with 10% fetal bovine serum (FBS; GE Healthcare Bio‐Sciences, Pittsburgh, PA), 10 U/ml recombinant mouse IFN‐γ (Thermo Fisher Scientific), and 300 μg/ml geneticin (Thermo Fisher Scientific) and maintained at the permissive temperature (33°C). To induce differentiation, podocytes were incubated in culture medium without IFN‐γ at 38°C (nonpermissive condition). Human MSCs and HUVECs were both acquired from the Bioresource Collection and Research Center, Food Industry Research and Development Institute, Hsinchu, Taiwan. The MSCs were cultured in α‐minimum essential medium (Thermo Fisher Scientific) supplemented with 20% FBS, 4 ng/ml basic fibroblast growth factor (PeproTech, Rocky Hill, NJ), 30 mg/ml hygromycin B (Thermo Fisher Scientific), and 200 mg/ml geneticin. HUVECs were grown in endothelial cell growth medium‐2 (EGM‐2; Lonza, Walkersville, MA).

### 5.2 Preparation of hybrid 3D cell spheroids

Fifty microliters of the prechilled 12% MC solution (Sigma‐Aldrich, St. Louis, MO) prepared in phosphate‐buffered saline was transferred into each well of 96‐well culture plates.^21^, ^23^ After incubation at 38°C for 30 min, 150 μl of EGM‐2 medium with a total of 4500 cells was added into each well of the MC hydrogel‐coated plates for subsequent incubation. For cell spheroids that were composed of multiple cell types, equal numbers of each cell type were used. The detailed cell types and cell numbers of each spheroid are as follows: P spheroids, 4500 podocytes; PE spheroids, 2250 podocytes + 2250 HUVECs; PM spheroids, 2250 podocytes  2250 MSCs; PME spheroids: 1500 podocytes + 1500 MSCs + 1500 HUVECs.

### 5.3 Characterization of hybrid 3D cell spheroids

The viability of cells within the spheroids was evaluated using a live/dead staining kit (Thermo Fisher Scientific) according to the manufacturer's instructions. For immunofluorescence staining, 3D cell spheroids were collected using a Pipetman and fixed in 4% paraformaldehyde for 15 min. After permeabilization with 0.1% Triton X‐100 and blocking with 5% normal goat serum (Vector Laboratories, Burlingame, CA) for 1 h, experimental samples were incubated with primary antibodies against CD31 (Agilent Technologies, Santa Clara, CA; #M0823; human specific), laminin (Abcam, Cambridge, MA; #ab11575), fibronectin (#ab6328), collagen IV (GeneTex, Hsinchu, Taiwan; #GTX26586), P‐cadherin (#GTX113648), VEGFA (#ab52917), HGF (#ab83760), IGF‐1 (#ab40657) or SDF‐1 (#GTX116092) at 4°C overnight. After three washes with PBS, the cells were incubated with Alexa Fluor 633‐conjugated secondary antibodies (Thermo Fisher Scientific) for 2 h at 37°C followed by counterstaining with 4',6‐diamidino‐2‐phenylindole (Thermo Fisher Scientific). Finally, the cell spheroids were mounted with a tissue‐clearing solution (FocusClear solution, CelExplorer, Hsinchu, Taiwan) and observed using a laser scanning confocal microscope (Carl Zeiss, Oberkochen, Germany).

To assess the migration of podocytes, cell spheroids were plated in culture plates and cultivated for 3 days. Fluorescence images were obtained, and the distances between the centers of spheroids and podocytes were measured for podocytes that traveled the longest distance using ImageJ software (NIH, Bethesda, MD).

For quantitative PCR, the total RNA of the experimental cells was extracted using TRIzol reagent (Thermo Fisher Scientific) and reverse‐transcribed into complementary DNA (cDNA) using a High‐Capacity cDNA Reverse Transcription Kit (Thermo Fisher Scientific) according to the instruction manual. Relative gene expression was determined using TaqMan gene expression assays for mouse *Gapdh* (Mm99999915_g1), *Synpo* (Mm03413333_m1), and *Vegfa* (Mm00437304_m1) and human *GAPDH* (Hs99999905_m1), *VEGFA* (Hs00900054_m1), and *HGF* (Hs00300159_m1) and the TaqMan Universal Master Mix II on a StepOnePlus Real‐Time PCR System (Thermo Fisher Scientific); each analysis was performed in triplicate.

For Western blot analysis, 3D cell spheroids were lysed by incubation with 200 μl of RIPA buffer (20 mM Tris–HCl, 1 mM EGTA, 150 mM NaCl, and 1% Triton X‐100) containing protease inhibitor cocktail tablets (Sigma‐Aldrich). Equal amounts of total protein extracts were denatured at 95°C for 10 min, subjected to sodium dodecyl sulfate‐polyacrylamide gel electrophoresis using an 8% acrylamide gel (Bio‐Rad Laboratories, Hercules, CA) and transferred to polyvinylidene difluoride membranes. The membranes were blocked with 5% skim milk for 1 h, followed by incubation with primary antibodies against fibronectin or laminin overnight. Finally, the membrane was probed with the HRP‐conjugated secondary antibody (Thermo Fisher Scientific) and detected using the Amersham ECL Select Western Blotting Detection Reagent (Cytiva, Marlborough, MA).

### 5.4 Animal model and hybrid 3D cell spheroid transplantation

All animal experiments were approved by the Institutional Animal Care & Utilization Committee of Linkou Chang Gung Memorial Hospital, and the care of the animals was in accordance with the Guidebook for the Care and Use of Laboratory Animals (third edition), published by the Chinese‐Taipei Society of Laboratory Animal Sciences in 2000. Transgenic mice (129S/SvEv‐Tg(Alb1‐Ren)2Unc/CofJ; stock #007853, Jackson Laboratory) that express renin under the modulation of a liver‐specific albumin promoter and exhibit kidney injury and proteinuria were employed to investigate the therapeutic efficacy of cell transplantation.^51^

For saline injection or cell transplantation, animals were anesthetized by inhalation of 2.5% isoflurane. An incision in the left flank was made to expose the left kidney, and 50 μl of plain saline or saline containing a PME suspension (3 × 10^5^ podocytes, 3 × 10^5^ MSCs, and 3 × 10^5^ HUVECs in total) or PME spheroids (200 PME spheroids; with the same cell composition and number as the suspension group) was injected slowly into two different sites of the cortex parenchyma of each kidney using a Hamilton syringe with a 26‐gauge needle. After 15 days, the kidneys were harvested and processed for histological analyses.

### 5.5 Kidney functional evaluation

The blood and urine of each experimental animal were collected preoperatively and before euthanization (Day 15). The BUN was determined using a commercial enzyme‐linked immunosorbent assay (ELISA) kit (Abcam; #ab83362). For urine samples, the concentrations of albumin and creatinine were measured using ELISA kits (both from Abcam; #ab108792 and #ab65340, respectively). The thus obtained data were used for calculating the level of urine albumin‐to‐creatinine ratio (UACR).

### 5.6 Histological analysis

The retrieved kidneys were fixed in 10% phosphate‐buffered formalin overnight, cryoprotected in 30% sucrose, embedded in optimal cutting temperature compound (Surgipath FSC22) and cut into 7‐μm‐thick sections using a cryostat microtome (Leica, Wetzlar, Germany). The sections were stained with hematoxylin and eosin, or probed with anti‐WT1 antibody before being visualized using 3,3'‐diaminobenzidine staining followed by counterstaining with hematoxylin. The number of podocytes in each glomerulus in 30 different fields per slide were counted in a blinded manner.

### 5.7 Statistical analysis

Data are expressed as the mean ± standard deviation. Statistical analyses were performed using GraphPad Prism software (version 8.2; San Diego, CA). For comparisons of two groups, an unpaired, two‐tailed Student's *t* test was used. One‐way ANOVA with Tukey's correction was employed for comparisons of three or more groups. Differences were considered to be significant at *p* < 0.05.

## AUTHOR CONTRIBUTIONS

**Chieh‐Cheng Huang:** Conceptualization; formal analysis; funding acquisition; project administration; resources; supervision; visualization; writing‐review and editing. **Chih‐Kuang Yeh:** Resources. **Yun‐Wei Lee:** Investigation. **Li‐Wen Hsu:** Methodology; resources. **Hsiang‐Hao Hsu:** Conceptualization; funding acquisition; project administration; resources; supervision; writing‐review and editing. **Ya‐Ting Jhuang:** Investigation; methodology; validation. **Li‐Chi Chen:** Investigation; methodology; validation. **Wen‐Yu Yang:** Conceptualization; formal analysis; investigation; methodology; validation; visualization; writing‐original draft. **Yu‐Jie Lin:** Investigation; methodology; validation. **Meng‐Yu Tsai:** Investigation; methodology. **Yi‐Ching Ko:** Investigation; methodology. **Pei‐Yu Hung:** Investigation; methodology.

## CONFLICT OF INTERESTS

The authors have declared no competing interest.

### PEER REVIEW

The peer review history for this article is available at https://publons.com/publon/10.1002/btm2.10212.

## Supporting information


**Figure S1** Representative live/dead images of 3D cell spheroids fabricated for 2 and 3 days. Scale bars, 100 μm.

**Figure S2** Representative fluorescence images of WT‐1‐stained kidney sections. Scale bars, 100 μm.

## Data Availability

The data that support the findings of this study are available from the corresponding author upon reasonable request.
