# Supplementary figures and images for: Injection of hybrid 3D spheroids composed of podocytes, mesenchymal stem cells, and vascular endothelial cells into the renal cortex improves kidney function and replenishes glomerular podocytes
Source: Bioeng Transl Med. 2021 Jan 21;6(2):e10212. doi: 10.1002/btm2.10212 (PMC8126810; doi:10.1002/btm2.10212)

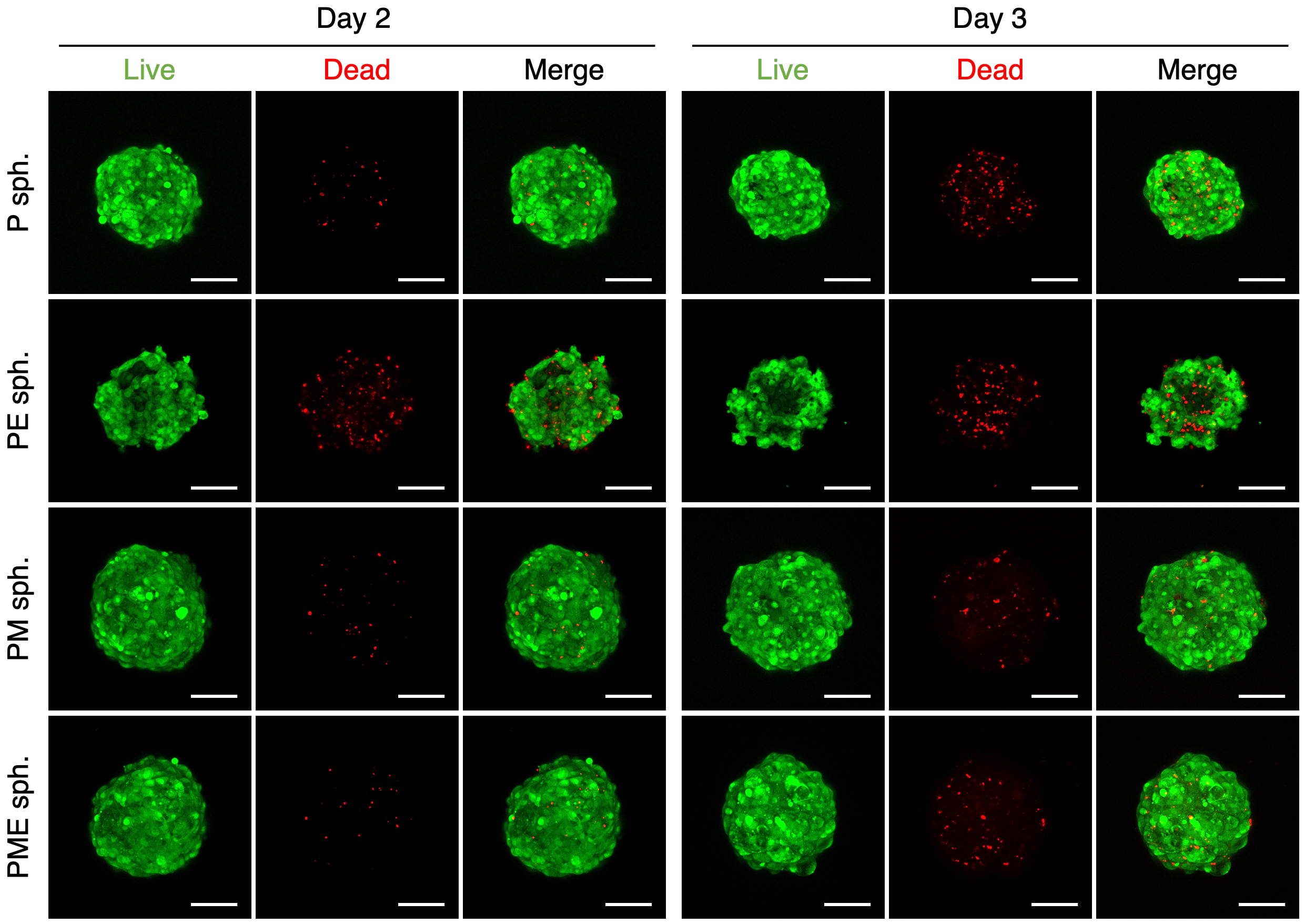

Supplement: Supplementary file 1 — Figure S1 Representative live/dead images of 3D cell spheroids fabricated for 2 and 3 days. Scale bars, 100 μm. [file BTM2-6-e10212-s001.tif]

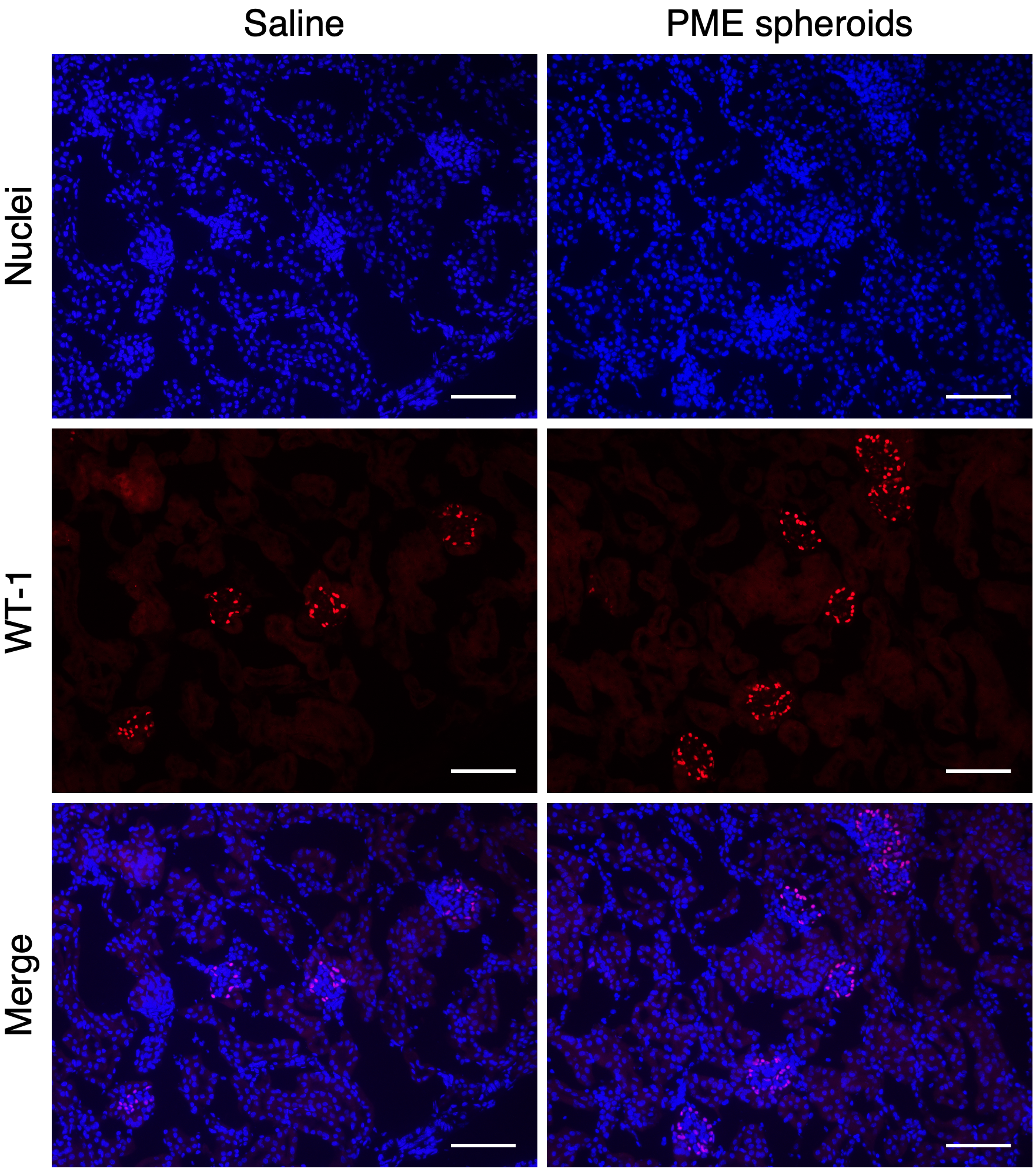

Supplement: Supplementary file 2 — Figure S2 Representative fluorescence images of WT‐1‐stained kidney sections. Scale bars, 100 μm. [file BTM2-6-e10212-s002.tif]
